# Functional Roles of Three Cutin Biosynthetic Acyltransferases in Cytokinin Responses and Skotomorphogenesis

**DOI:** 10.1371/journal.pone.0121943

**Published:** 2015-03-24

**Authors:** Lei Wu, Zhao-Yang Zhou, Chun-Guang Zhang, Juan Chai, Qin Zhou, Li Wang, Eva Hirnerová, Michaela Mrvková, Ondřej Novák, Guang-Qin Guo

**Affiliations:** 1 MOE Key Laboratory of Cell Activities and Stress Adaptations, School of Life Sciences, Lanzhou University, Lanzhou, China; 2 Laboratory of Molecular and Cell Biology, Hebei Normal University, Shijiazhuang, China; 3 Laboratory of Growth Regulators, Centre of the Region Haná for Biotechnological and Agricultural Research, Institute of Experimental Botany ASCR & Faculty of Science, Palacký University, Šlechtitelů 11, Olomouc, Czech Republic; Instituto de Biología Molecular y Celular de Plantas, SPAIN

## Abstract

Cytokinins (CKs) regulate plant development and growth via a two-component signaling pathway. By forward genetic screening, we isolated an *Arabidopsis* mutant named *grow fast on cytokinins 1 * (*gfc1*), whose seedlings grew larger aerial parts on MS medium with CK. *gfc1* is allelic to a previously reported cutin mutant *defective in cuticular ridges* (*dcr*). *GFC1/DCR* encodes a soluble BAHD acyltransferase (a name based on the first four enzymes characterized in this family: Benzylalcohol O-acetyltransferase, Anthocyanin O-hydroxycinnamoyltransferase, anthranilate N-hydroxycinnamoyl/benzoyltransferase and Deacetylvindoline 4-O-acetyltransferase) with diacylglycerol acyltransferase (DGAT) activity *in vitro* and is necessary for normal cuticle formation on epidermis *in vivo*. Here we show that *gfc1* was a CK-insensitive mutant, as revealed by its low regeneration frequency *in vitro* and resistance to CK in adventitious root formation and dark-grown hypocotyl inhibition assays. In addition, *gfc1* had de-etiolated phenotypes in darkness and was therefore defective in skotomorphogenesis. The background expression levels of most type-A *Arabidopsis Response Regulator* (*ARR*) genes were higher in the *gfc1* mutant. The *gfc1-*associated phenotypes were also observed in the cutin-deficient *glycerol-3-phosphate acyltransferase 4/8* (*gpat4/8*) double mutant [defective in glycerol-3-phosphate (G3P) acyltransferase enzymes *GPAT4* and *GPAT8*, which redundantly catalyze the acylation of G3P by hydroxyl fatty acid (OH-FA)], but not in the cutin-deficient mutant *cytochrome p450*, *family 86*, *subfamily A*, *polypeptide 2/aberrant induction of type three 1* (*cyp86A2/att1*), which affects the biosynthesis of some OH-FAs. Our results indicate that some acyltransferases associated with cutin formation are involved in CK responses and skotomorphogenesis in *Arabidopsis*.

## Introduction

Cytokinins (CKs) are N^6^-substituted adenine derivatives that play essential roles in regulating plant growth and development, including shoot initiation and growth, leaf senescence, and photomorphogenesis [1–4]. The predominant CKs in higher plants are isopentenyladenine (iP), *trans*-zeatin (tZ) and dihydrozeatin (DHZ) [5]. *In planta* CK homeostasis is regulated by a balance between biosynthesis and catabolism: the rate-limiting step of CK biosynthesis is catalyzed by enzymes encoded by the ATP/ADP- isopentenyltransferase (IPT) gene family [6], and its degradation is regulated by the activity of seven CK oxidase/dehydrogenases (CKX1–7) [7].

Plants respond to CKs through a multi-step phosphorelay system, consisting of sensor histidine kinases (HK), histidine phosphotransfer proteins (HP), and effector response regulators (RR) (two-component system) [2,3,8–10]. So far three CK receptors, AHK2, AHK3 and AHK4/CYTOKININ RESPONSE 1 (CRE1)/WOODEN LEG (WOL) have been identified [3,11–14] with overlapping as well as specific functions in regulation of shoot, root, and embryo growth and of senescence [15]. In *Arabidopsis*, the phosphorelay initiated from the receptors incorporates both AHPs and ARRs. AHPs mediate the transfer of phosphoryl groups from AHKs to type-B ARRs [16], which function as transcription factors [17–19]. Among the transcriptional targets of the type-B ARRs are type-A ARRs, the negative regulators of the CK signal pathway [20–22]. Both type-A and type-B ARRs are partially redundant regulators in CK signaling [20,23–27]. A third family, called type-C ARRs, have similar structures to those of the type-A ARRs, but their expression is not induced by CK [28,29]. The role of the type-C ARRs in CK signaling remain unclear [28].

There is extensive crosstalk between CK and other plant hormone signaling [15]. The antagonistic interaction between CK and auxin to control shoot and root development is well-known. Intricate crosstalk between CK and ethylene regulates hypocotyl elongation, in which a major portion of CK action is through ethylene, mainly by stimulating its biosynthesis at the post-transcriptional level [30,31].

In lipid metabolism, acylation is necessary for synthesizing various storage/structural lipids, such as triacylglycerol (TAG) and membrane lipids [32]. Acylation is also required for synthesizing many kinds of secondary metabolites and extracellular matrix materials in plants [33], such as cutin for the epidermal cuticle layer, which is synthesized through a complex process that consists of fatty acid (FA) synthesis, FA activation into acyl-coenzyme A (CoA), ω- and/or in-chain oxygenation, sn-2 monoacylglycerol (MAG) synthesis, monomer/oligomer transport out of the cell to the surface and polymerization into cutin polyester [34]. There is also some evidence that acylation plays roles in regulating hormone levels or activity, either through direct modification [35] or the acylation of amino acids [36]. However, roles of acyltransferases in CK action have not yet been reported.

In the process of screening CK mutants in *Arabidopsis*, we isolated a CK-insensitive mutant named *gfc1*, which also had some photomorphogenic phenotypes in darkness. *gfc1* is allelic to the previously reported cuticle mutant *dcr*. The *GFC1/DCR* gene encodes a soluble enzyme of the BAHD acyltransferase family that is necessary for normal cuticle formation on epidermis *in vivo* [37] and has diacylglycerol acyltransferase (DGAT) activity *in vitro* [38]. We showed that the cuticle-defective, double mutant of the genes encoding G3P acyltransferases GPAT4 and GPAT8, but not a mutant of the FA ω-hydroxylase *CYP86A2* gene, also led to *gfc1*-associated phenotypes. Our results indicate that some acyltransferases involved in cutin assembly are important for CK responses and skotomorphogenesis in *Arabidopsis*.

## Materials and Methods

### Plant material and growth conditions

Approximately 40,000 *Arabidopsis* (Col-0) T-DNA insertion lines (stock numbers CS76502, CS76504, CS76506 and CS76508) were purchased from the Arabidopsis Biological Resource Center (http://abrc.osu.edu/). For all lines except *ahk3–1* (Ws-2), *Arabidopsis* accession Col-0 was used as WT. Mutant and transgenic lines used in this study have been described previously: *pCYCB1*:*GUS* marker line [39], *ARR-OX* lines (homozygous transgenic plants in [40]), *ahk3–1* (N6562), *gfc1–2/dcr-2* (salk_128228c), *gpat4* (salk_106893), *gpat8* (salk_035914), *cyp86A2* (salk_128714c), *phyA* (N6223, *phyA-211*) and *phyB* (N6217, *phyB-9*) were purchased from ABRC.

Seeds were treated at 4°C for 2 days in water. Before inoculation on MS, seeds were sterilized with 0.1% w/v HgCl_2_. For growth under long day condition, germination and plant growth took place with a 16-h light (60–70 μmol m^−2^ s^−1^) / 8-h dark cycle. For growth in darkness, germination took place under white light (60–70 μmol m^−2^ s^−1^) at 22°C for 3 h. Seeds were plated on MS (pH 5.8) with agar at either 1.0% (w/v) (vertical plate) or 0.8% (w/v) (horizontal plate) and 1% (w/v) sucrose and grown at 22°C.

To introduce a reporter gene into the mutant, *pCYCB1*:*GUS* was crossed with *gfc1–1* and double homozygotes were identified in the F_3_ generation.

For DIC, 5 DAG seedlings were grown on vertical plate under long day condition.

For adventitious root formation assays and callus induction assays, 11 DAG seedlings grown on horizontal plate under long day condition were used.

For Toluidine blue-O staining, 3-week-old plants grown in soil under long day condition were used. Relative humidity was approximately 60%.

### Gene cloning and sequence analysis

Inverse-PCR (I-PCR) was adopted to clone the mutant gene. Genomic DNA was digested completely with HindIII and ligated with T4 DNA ligase. Two rounds of PCR were performed with two sets of nested primers, LBb_1_ / Z4 and LBb_1_ / Z3 (S1 Appendix). The PCR fragments were subcloned and sequenced. The downstream flanking sequence was amplified by PCR with LBb_1.3_ and 3362-F primers (S2 and S3 Appendix). Flanking sequences (S1 Appendix) were used for designing gene-specific primers to determine hetero-/homozygosity and co-segregation (S3 Appendix).

### Phenotype characterization

For DIC, roots were cleared by the chloral hydrate method as described by Inagaki et al. [41].

To measure root length and fresh weight of shoots, seedlings were germinated and grown on vertical MS plates under long day condition with different concentrations of tZ (Sigma, http://www.sigmaaldrich.com), thidiazuron (TDZ, Sigma), isopentenyladenine (iP, Sigma), 6-benzyladenine (6-BA, Sigma) or kinetin (KT, Sangon, http://www.sangon.com) at 7 DAG.

For hypocotyl inhibition assays, seedlings were germinated and grown on vertical MS plates in darkness with different concentrations of CKs, ACC (Sigma), IAA (Sigma) and GA3 (Sigma) at 5 DAG.

For adventitious root formation assays and callus induction assays, the methods were performed according to [42].

For light-induced analysis, all experiments involving blue, red, or far-red light illumination were performed under 16-hr L/8-hr D light conditions in an E-30 LED growth chamber (Percival, Boone, IA, http://www.percival-scientific.com) with the blue (k_max_ 469 nm, PPFD = 50 μmol m^−2^ s^−1^), red (k_max_ 680 nm, PPFD = 50 μmol m^−2^ s^−1^), or the far-red (k_max_ 730 nm, PPFD = 50 μmol m^−2^ s^−1^) diodes at 22 °C.

For Toluidine blue-O staining, plants were immersed for 5 min in 0.05% Toluidine blue-O (Sigma) and rinsed with water at room temperature.

### Molecular complementation and *pGFC1*:*GUS* transgenic plants

A 2.2-kb promoter sequence was amplified using PGFC1-F2 / PGFC1-R primers (S2 Appendix) and sub-cloned into a modified pCAMBIA1300 binary vector harboring a GUS gene to generate a promoter:GUS reporter gene construct.

The 1.6-kb full-length cDNA fragment of *GFC1* gene was amplified by RT-PCR using GFC1-F / GFC1-R primers (S2 Appendix) and ligated downstream of the 2.2-kb *GFC1* promoter to construct *pGFC1*:*GFC1* in a pCAMBIA1300 vector. All amplified DNA fragments were confirmed by sequencing, and the constructed binary vectors were introduced into either WT plants (for *pGFC1*:*GUS*) or *gfc1–1* plants (for *pGFC1*:*GFC1*) by an *Agrobacterium tumefaciens-*mediated (strain GV3101) floral-dip transformation method [43]. Primary transformants were isolated on MS containing 25mg/L hygromycin (Sigma) and transferred to soil to grow to maturity.

### Histochemical GUS assay

Seedlings containing *GUS* marker were subjected to various treatments and then were incubated in 1 mmol/L X-gluc (5-bromo-4-chloro-3-indolyl-β-D-glucuronide) and 50 mmol/L potassium phosphate buffer, pH 7.5, with 0.1% (v/v) Triton X-100 for GUS staining as described by Jefferson [44].

### RNA preparation and expression analysis

*Arabidopsis* seedlings were immediately frozen in liquid nitrogen, and stored at-80 °C. RNA was isolated using Trizol (Sangon) and reverse-transcribed using a reverse transcription kit (DRR047A) (Takara, http://www.takara-bio.com/). Quantitative RT-PCR was performed in a Bio-Rad CFX96 Real-time System (Bio-Rad, http://www.bio-rad.com) using Power SYBR green chemistry (DRR081A) (Takara). Primer sequences used are listed in S2 Appendix.

For CK-induced type-A *ARR* expression, seeds were germinated on MS plates and grown for 7 days. Treatments were carried out by incubating seedlings in liquid 1/2 MS culture medium containing 1% sucrose and supplemented with 10 μM tZ for 30 min [45].

For CK-induced *GFC1* expression, we treated seedlings with 1 μM tZ for 30 min.

### Endogenous CK measurement

Endogenous levels of CKs were determined by LC-MS/MS methods according to [46] with modifications. Briefly, ice-cold modified Bieleski buffer (methanol/water/formic acid, 15/4/1, v/v/v; [47]) and two SPE columns (C18 column—500 mg/Applied Separations, and MCX column—30 mg/Waters; [48]) were used to extract 100 mg seedlings (7 DAG). To each extract the stable isotope-labeled CK internal standards (0.5 pmol of CK bases, ribosides, *N*-glucosides, 1 pmol of *O*-glucosides and nucleotides) were further added as a reference. Analytes were eluted by two-step elution using a 0.35 M NH_4_OH aqueous solution and 0.35 M NH_4_OH in 60% (v/v) MeOH solution. All samples were then evaporated under vacuum at 37°C to dryness.

Purified samples were analyzed by the LC-MS/MS system consisting of an ACQUITY UPLC System (Waters) and Xevo TQ-S (Waters) triple quadrupole mass spectrometer. Quantification was obtained using a multiple reaction monitoring (MRM) mode of selected precursor ions and the appropriate product ion. The linear range was over at least five orders of magnitude with a correlation coefficient of 0.9989–0.9998. For each mutant, four independent biological replicates were performed.

### Measurement of ethylene production

Ethylene level was measured by gas chromatography as described [49]. *Arabidopsis* seedlings (5 DAG) grown on MS or MS with 10 μM tZ in darkness were used. Seedlings (100 mg) were placed in a 2 ml vial (Agilent Technologies, http://www.agilent.com) and sealed. After 2 h, 0.5 ml samples of the air inside the vials were used for the determination of ethylene production (Varian 450-GC, http://www.varian.com). For each treatment, four independent biological replicates were performed.

## Results

### Mutant isolation and molecular identification of *GFC1* gene

**Fig 1:**
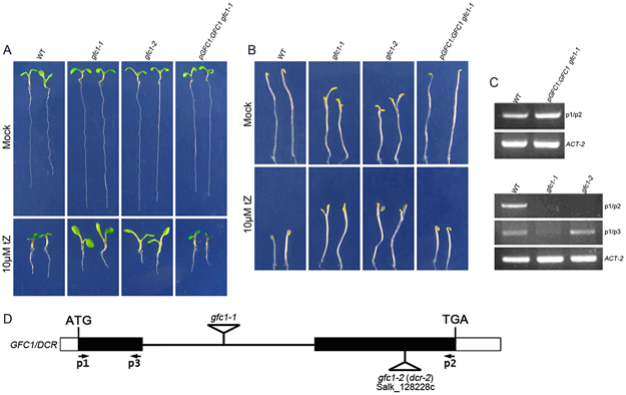
*gfc1* mutants and molecular complementation. (A, B) Phenotypes of WT, *gfc1* mutant lines, and *pGFC1*:*GFC1 / gfc1–1* under long day conditions (A) or in darkness (B). (C) RT-PCR analysis (30 cycles) of the *GFC1* transcript in *pGFC1*:*GFC1 / gfc1–1*, *gfc1*, and *ACT-2* was used as a control. (D) *GFC1* gene structure and location of T-DNA insertions. p1, p2, p3 are primers for RT-PCR (S2 Appendix).

To isolate genes potentially involved in CK responses, we used a forward genetics approach. Approximately 40,000 T-DNA insertion lines [50] were screened to isolate mutants with altered responses to CK in terms of root/shoot growth. The *gfc1–1* mutant was identified by the significantly large aerial components of seedlings grown on MS containing 10 μM tZ (*grow fast on cytokinins 1*, *gfc1*, Fig. 1A). In darkness, *gfc1–1* mutants had short hypocotyls, which were insensitive to exogenous tZ, an opened apical hook, and over-grown cotyledons—the so-called de-etiolated-like phenotypes (Fig. 1B, details see below). Furthermore, *gfc1–1* mutant had higher percentage of non-germinating seeds than WT (S2 Fig.).

To clone the *GFC1* gene, we first back crossed the *gfc1–1* mutant into WT for two generations. F_1_ lines showed a WT-like phenotype and F_2_ lines segregated for WT and *gfc1* phenotypes in a 3:1 ratio (588:189), indicating that *gfc1* is a single recessive mutation. Inverse PCR amplification of T-DNA flanking sequences (S1 Appendix) identified a T-DNA insertion in the only intron (1289 bp downstream of ATG) of the gene At5g23940, which has been reported to encode a soluble BAHD acyltransferase essential for normal cuticle formation on epidermis [37]. Additional sequence analysis of regions surrounding the T-DNA insertion site indicated no sequence alterations in the adjacent regions (S1 Appendix). Co-segregation analysis of the F_2_ backcross progeny showed a linkage between the *gfc1* mutant phenotype and the insertion in the *GFC1* gene.

In order to verify that the phenotype of the *gfc1–1* mutant indeed originated from a lesion in the At5g23940 gene, we identified an additional mutant allele, salk_128228c, with the T-DNA insertion at the second exon (2904 bp downstream of ATG) (Fig. 1D). Homozygous salk_128228c seedlings displayed the same *gfc1* phenotypes (Fig. 1A- C) and this allele was named *gfc1–2*, (*dcr-2* in [37]). We also observed the cutin-associated phenotypes in *gfc1* mutants (see below). When the homozygous *gfc1–1* plants were transformed with the *GFC1* genomic sequence under the control of the *GFC1* promoter (*pGFC1*:*GFC1*), its mutant phenotypes were fully rescued when observed in T_2_ homozygous transgenic plants (Fig. 1A-C).

### *gfc1* mutant exhibited increased root meristematic activity

**Fig 2:**
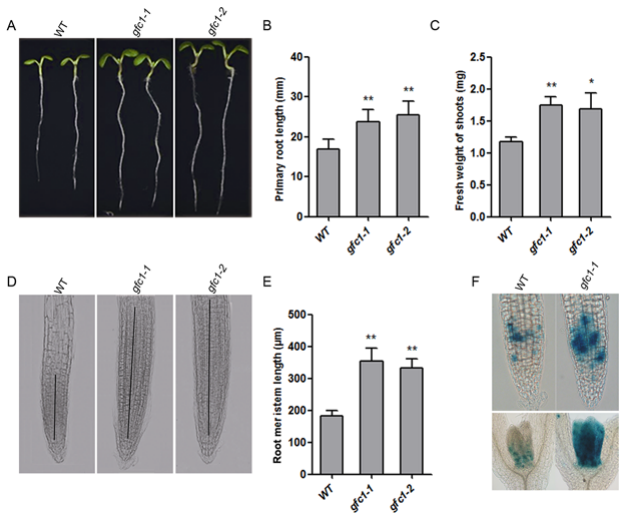
Comparison of phenotypes between WT and *gfc1* mutants under long day conditions. (A-C) Seedlings 7 days after germination (DAG) (A), and measurement of primary root length (A, B) and fresh weight of shoots (C). (D, E) Meristematic Zone (MZ) of roots 5 DAG. (F) Expression pattern of *pCYCB1*:*GU*S in seedlings 7 DAG. All the data are mean of three biological replicates, n = 50 (B, C), n = 25 (E). Error bars indicate standard deviation (SD). *P <0.05 and **P <0.01 (Student’s t-test).

When grown on vertical MS plates, *gfc1* seedlings showed a variety of phenotypes. The primary root length of *gfc1* seedlings was significantly longer than that of WT (Fig. 2A and 2B). The fresh weight of shoots was also significantly increased in the two mutants (Fig. 2C).

To determine whether the increased primary root growth in *gfc1* mutants is a result of increased root meristematic activity or not, we compared the root structures of *gfc1* mutants with WT. DIC images showed that the root meristem zone length (MZ, extending from the quiescent center (QC) to the first elongated cell) [41] was significantly increased in *gfc1* when compared with that in WT (Fig. 2D and 2E). We introduced *pCYCB1*:*GUS*, a cell cycle marker for G2/M transition [51,52] into the *gfc1–1* mutant. Consistent with meristem phenotype, the expression of *pCYCB1*:*GUS* gene was greatly increased in *gfc1–1* seedlings (Fig. 2F).

### *gfc1* seedlings have altered responses to CKs

**Fig 3:**
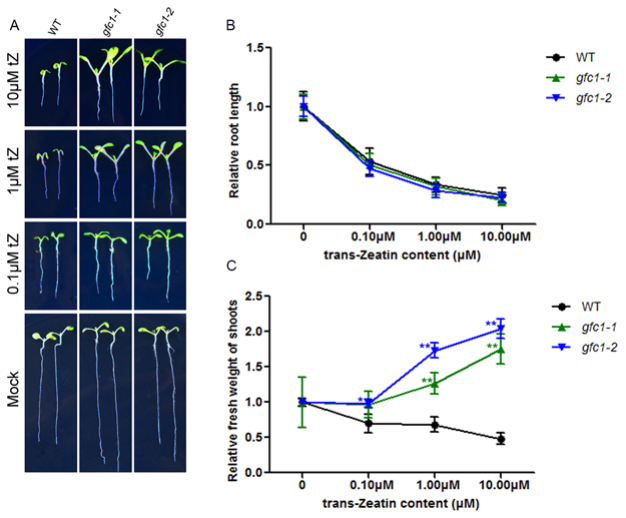
Seedlings were grown on MS with different concentrations of tZ at 7 DAG. (A) tZ-induced phenotypes. Data in (B, C) are mean of three biological replicates, n = 25 (B), 50 (C). Error bars indicate SD. Asterisks indicate statistically significant difference in the mutant lines versus the WT in a student’s t-test (*P <0.05 and **P <0.01).

We examined the sensitivity of *gfc1* to CK in several assays, focusing on CK-mediated growth and development. External application of CK significantly inhibits the growth of WT seedlings. When treated with tZ, root elongation of the *gfc1* seedlings was normally inhibited (Fig. 3A and 3B). While tZ inhibited WT shoot growth in terms of fresh weight (Fig. 3C), it strikingly induced significant fresh weight increases in the *gfc1* shoots (Fig. 3A and 3C). Other CKs had similar effects on *gfc1–1* seedlings (S3 Fig.). However, unlike CKs, other hormones or their biosynthetic precursors, including the auxin indole-3-acetic acid (IAA), gibberellic acid (GA_3_), and the ethylene precursor 1-aminocyclopropane-1-carboxylic acid (ACC), failed to induce significantly differential responses between *gfc1* and WT (S4 Fig.).

### Callus culture and adventitious root formation

**Fig 4:**
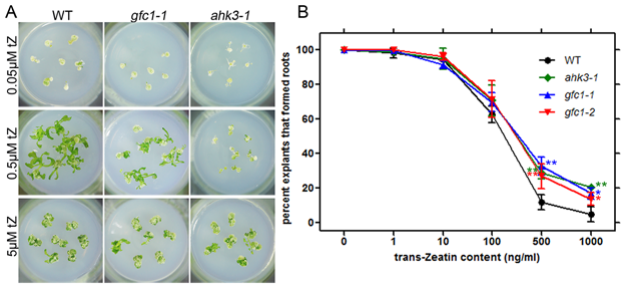
CK sensitivity of callus *in vitro*. (A) Callus formation and greening/shoot initiation from hypocotyl segments of WT, *gfc1–1* and *ahk3–1*. (B) CK inhibition of adventitious root formation. All the data are mean of three biological replicates, n = 30–40. Error bars indicate standard deviation (SD). Asterisks indicate statistically significant differences in the mutant lines versus the WT in a student’s t-test (*P <0.05 and **P <0.01).

CKs are used to stimulate cell division and greening/shoot initiation of callus tissue [53]. On media containing 0.05 μM 2,4-D with different concentrations of tZ, the CK-induced cell division and greening of hypocotyl-derived calli were reduced in both *ahk3–1* and *gfc1–1* mutants when compared to WT, with *ahk3–1* showing the least response (Fig. 4A).

CKs normally inhibit adventitious root formation near the cut end of hypocotyls [54]. The *gfc1* mutants were less sensitive to CKs in the adventitious root formation assay, similar to the *ahk3–1* mutant (Fig. 4B).

### Skotomorphogenesis and CK treatment of *gfc1* seedlings in darkness

**Fig 5:**
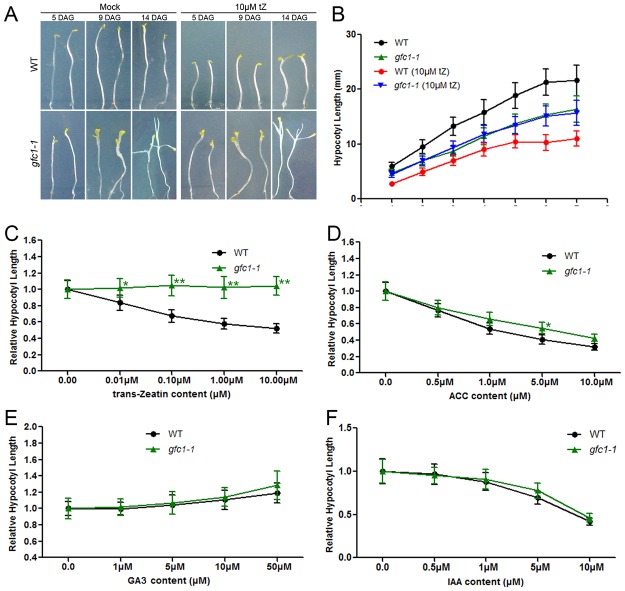
The dark-grown phenotypes of *gfc1–1* seedlings were grown on MS with different hormones. Data in (C-F) are mean of three biological replicates with 5 DAG seedlings, n = 25. Error bars indicate SD. Asterisks indicate statistically significant differences between the mutant lines and the WT in a student’s t-test (*P <0.05 and **P <0.01).

CK can induce dark-grown seedlings to develop some photomorphogenic or de-etiolation characteristics, including reduced hypocotyl growth, apical hook opening, cotyledon expansion, and the induction of leaf development (Fig. 5A) [55–57]. The *gfc1* mutant was observed to have those obvious de-etiolation-like phenotypes without CK (Fig. 5A). However, *gfc1* mutant seedlings displayed completely normal photomorphogenic responses to red light (Rc), blue light (Bc) and far red light (FRc) (S5 Fig.), suggesting that *gfc1* is defective only in skotomorphogenesis.

In darkness, the *gfc1* mutant exhibited short hypocotyls compared with WT (Fig. 5A and 5B). They showed overgrown cotyledons and formed the first true leaves 14 days after germination (Fig. 5A). When treated with CKs, the hypocotyl length in dark-grown WT seedlings decreased significantly, while there was no significant decrease in hypocotyl length in *gfc1* mutant (Fig. 5A-C and S3 Fig.). However, *gfc1* showed WT-like responses to the ethylene biosynthesis precursor ACC (Fig. 5D) and to IAA (Fig. 5F), both of which suppress the hypocotyl elongation of both *gfc1* and WT seedlings in darkness [58]. GA_3_ slowly stimulates rather than inhibits hypocotyl elongation in darkness. The *gfc1–1* mutant showed a WT-like response to application of GA_3_ (Fig. 5E).

### CK-response genes: Elevated basal expression and reduced CK induction

**Fig 6:**
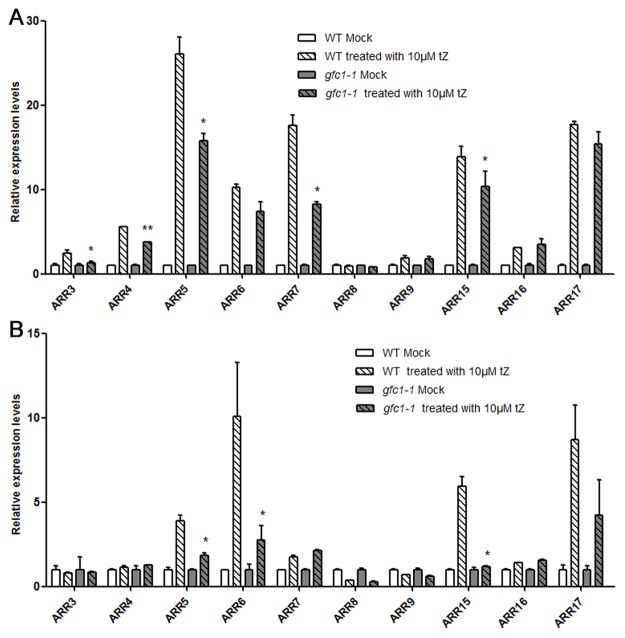
Type-A *ARR* gene expression levels after 30 min treatment with exogenous tZ. WT and *gfc1–*1 seedlings were grown under long day condition (A) or in darkness (B) for 7 days. Expression levels were normalized to the *ACT-8* transcripts. Mock treated WT or *gfc1–1* were used as controls. Relative expression values = Treatment/Mock. Data are mean of three biological replicates, n = 3. Error bars indicate SD. *P <0.05 and **P <0.01 (Student’s t-test) indicate significant between ‘WT treated with 10 μM tZ’ and ‘*gfc1–1* treated with 10 μM tZ’.

CKs induce the transcription of type-A *ARRs* in *Arabidopsis* [22]. To determine whether the induction levels of these primary CK response genes were compromised in *gfc1–1*, real-time quantitative PCR (qRT-PCR) analysis was performed on RNA prepared from WT and *gfc1–1* mutants. Exogenous CK treatment of *gfc1–1* did up-regulate the transcription of type-A *ARRs*, although the relative induction levels were lower than those in WT (Fig. 6), indicating that the mutant has a reduced CK response.

**Fig 7:**
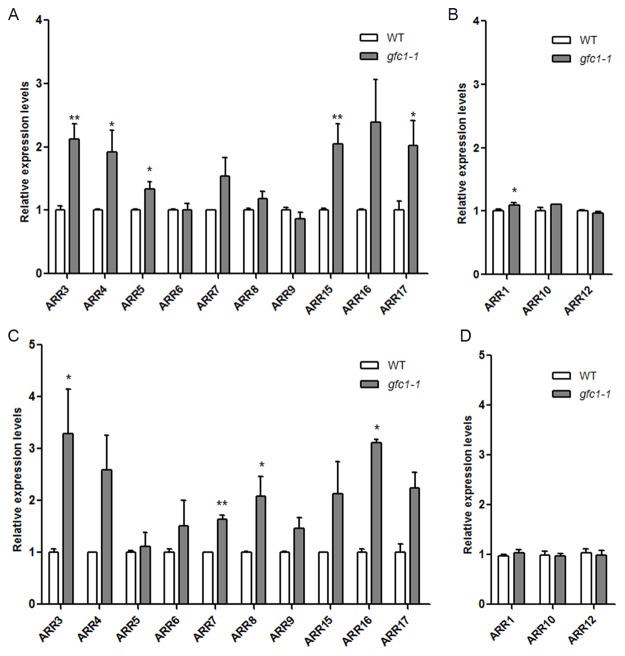
Comparison of the expression levels of *type-A ARR* genes (A, C) and *type-B ARR* genes (B, D) between WT and *gfc1–1*. Seedlings were grown under long day condition (A, B) or in darkness (C, D) for 7 days. Expression levels were normalized to the *ACT-8* transcripts. WT was used as a control. Data are mean of three biological replicates, n = 3. Error bars indicate SD, *P <0.05 and **P <0.01 (Student’s t-test).

**Fig 8:**
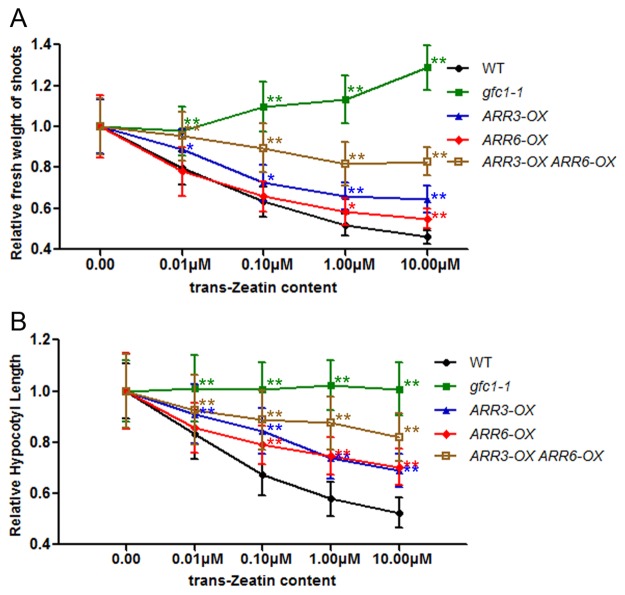
Comparison of CK-induced phenotypes in WT, *gfc1–1* and *ARR-OX* lines. (A) Relative fresh weight of the shoots of seedlings grown on MS with different concentrations of tZ at 7 DAG under long-day condition. (B) Relative hypocotyl length of seedlings grown on MS with different concentrations of tZ in darkness at 5 DAG. Data are mean of three biological replicates, n = 12 (A), n = 25 (B). Error bars indicate SD. Asterisks indicate statistically significant difference in the mutant lines versus the WT in a student’s t-test (*P <0.05 and **P <0.01).

Comparison of the basal expression levels of these *ARR*s in WT and *gfc1–1* on MS without CK showed that most type-A *ARR*s displayed significantly higher levels in *gfc1–1* (Fig. 7A and 7C). Overexpression of type-A *ARR*s is reported to lead to decreased CK sensitivity [27,40]. In *ARR15-OX* plant, the CK induction levels of *ARR4* and *ARR7* were significantly decreased [27]. Basal up-regulation of some type-A *ARR*s and their relatively reduced responses to CK may be only part of the reason for *gfc1* CK-insensitivity (Fig. 8). Unlike type-A *ARR*s, the expressions of type-B *ARR*s (*ARR1*, *10* and *12*) were not significantly different between *gfc1–1* and WT (Fig. 7B and 7D).

### Defective *GPAT*s for cutin biosynthesis can phenocopy *gfc1*

*GFC1/DCR* has been shown to be essential for the assembly of cutin polyesters [37] and encodes a soluble BAHD acyltransferase that can catalyze the incorporation of dicarboxylic fatty acids (DFA) or OH-FAs into diacylglycerol to form TAG *in vitro* [38]. This raised the question of whether other enzymes in the cutin biosynthesis pathway, such as GPATs for sn-2 MAG synthesis and some P450 monooxygenases for FAs hydroxylation [34], have the same or similar roles in CK response.

**Fig 9:**
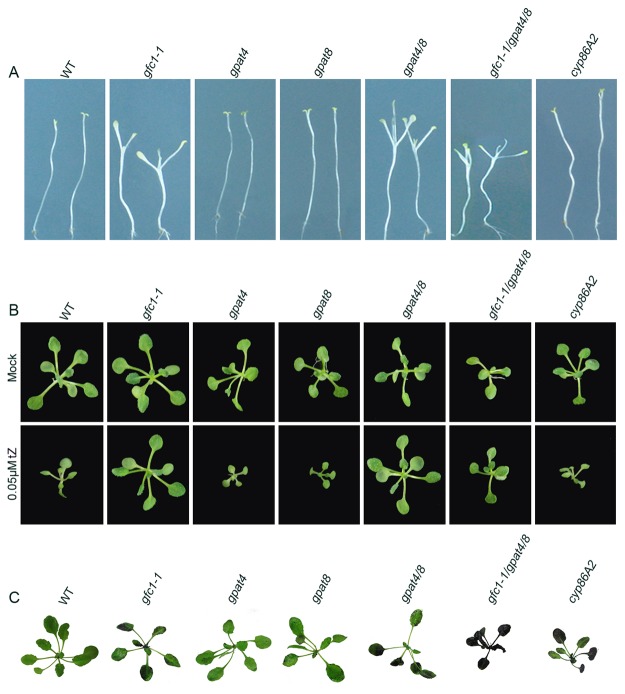
Comparison of phenotypes of WT and various cutin-defective mutants. (A) Darkness, 14 DAG. (B) Long day conditions, 14 DAG. (C) Cuticle permeability to Toluidine blue-O.

It has been reported that GPAT4 and GPAT8 are functionally redundant and that a *gpat4/8* double mutant is defective in cutin biosynthesis [59]. Strikingly, we found that the *gpat4/8* double mutant had the same or similar dark-grown and CK-induced phenotypes as *gfc1* (Fig. 9A-B and S6A-D Fig.). Significantly, the phenotypes were more severe in the *gfc1–1/gpat4/8* triple mutants (Fig. 9A-B and S6A-D Fig.).

Several cytochrome P450 monooxygenases, such as CYP86As, can catalyze the ω-hydroxylation of fatty acyl chains, which are the major cutin monomers. Similar to *gfc1 and gpat4/8*, the *cyp86A2/att1* mutant has epidermal cuticle defects with increased permeability as revealed by toluidine blue-O staining (Fig. 9C) [59,60]. In contrast to *gfc1 and gpat4/8*, however, when treated with CK or grown in darkness, the *cyp86A2*/*att1* mutant failed to show *gfc1*-like phenotypes (Fig. 9A-B and S7A-D Fig.), suggesting that the *gfc1* phenotypes were not necessarily related to general cuticle defects in epidermis.

### Tissue distribution of *GFC1* expression

**Fig 10:**
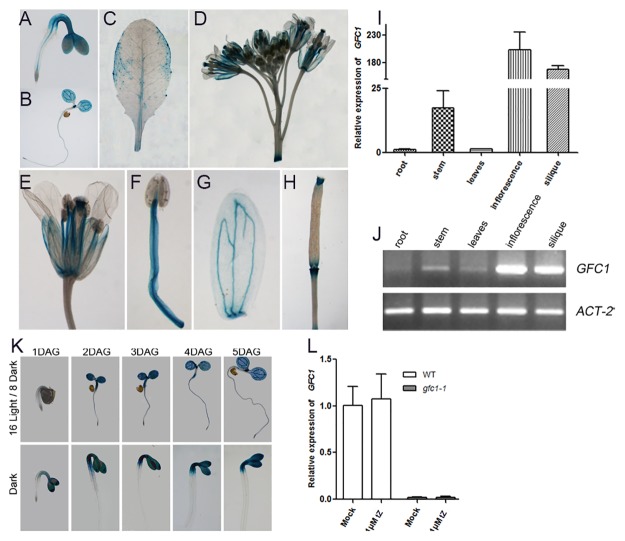
Expression analysis of *GFC1*. Expression pattern of GUS reporter gene in *pGFC1*:*GUS* transgenic *Arabidopsis* (A-H, K). (A) 1 DAG; (B) 5 DAG; (C) Rosette leaf; (D) Inflorescence; (E) Flower; (F) Stamen; (G) Sepal and (H) Silique. (K) Seedlings were grown under long day conditions or in darkness. (I, L) Expression analysis of *GFC1* by qRT-PCR, *ACT-8* was used as an internal control. Data are means of three biological replicates, n = 3. Error bars indicate SD. (J) Expression analysis of *GFC1* by RT-PCR (30 cycles). *ACT-2* was used as a control.

The expression of *GFC1* was followed by introducing a *GFC1* promoter-GUS reporter fusion (*pGFC1*:*GUS*) into WT. Staining of the GUS activity in independent transgenic lines in T_4_ progenies showed that the *GFC1* promoter was highly active in cotyledon and the apical hook (Fig. 10A-B and 10K) in seedlings. In mature plants, strong GUS staining was observed in the upper stem, inflorescences, and siliques (Fig. 10C-J), results which are similar to those reported for DCR in [37]. Notably, the expression level of *GFC1* is not elevated by exogenous tZ (Fig. 10L). These results are consistent with the gene expression data retrieved from the GENEVESTIGATOR using Meta-Analyzer.

### Endogenous CKs in *gfc1–1* and *gpat4/8* mutants

In *Arabidopsis*, CKs can induce de-etiolation in WT plants [55,57,61,62]. To know if the de-etiolation and altered CK-response phenotypes in *gfc1* and *gpat4/8* mutants were associated with any changes in CK metabolism, we compared the levels of various endogenous CKs between 7DAG seedlings of WT and the mutants by LC-MS/MS. The results showed no significant differences between the two mutants and the WT in either total CK content or each of the total tZ/cZ/DHZ/iP types (Table 1). Although both mutants had higher levels of active tZ-type cytokinins than WT, they showed reduced levels in free iP but increased levels in iPRMP. Some of these differences were not statistically significant. iPR was significantly decreased in *gfc1–1*, but increased in *gpat4/8*. Because of the strong and very similar CK-associated *gfc1* phenotypes between these two mutants, the CK quantification results revealed no obvious link between CK homeostasis and the *gfc1* phenotype.

**Table 1:** CK levels in 1g of extracted tissue (pmol/g, Mean±SD).

Sample	tZ	tZR	tZOG	tZROG	tZ7G	tZ9G	tZRMP	Total tZ-type CKs
**Col-0**	0.37±0.04	0.53±0.08	5.45±0.74	0.67±0.11	14.54±1.24	5.72±0.45	1.29±0.22	28.57±2.30
***gpat4/8***	0.50±0.05*	0.79±0.11*	7.34±0.62*	1.07±0.19*	13.55±1.85	5.05±0.23	2.43±0.57*	30.73±2.97
***gfc1–1***	0.42±0.05	0.64±0.10	7.17±1.50	1.22±0.21**	14.32±0.93	6.09±0.94	2.71±0.64*	32.57±2.79

Sample	cZ	cZR	cZOG	cZROG	cZ9G	cZRMP	Total cZ-type CKs
**Col-0**	0.08±0.01	1.24±0.26	1.46±0.17	4.19±0.48	0.21±0.03	7.02±1.24	14.21±1.75
***gpat4/8***	0.10±0.01	0.81±0.12*	2.84±0.19***	5.39±0.33*	0.27±0.02*	7.15±0.53	16.56±0.61
***gfc1–1***	0.13±0.02*	0.56±0.06**	1.07±0.10*	3.44±0.54	0.22±0.03	8.23±1.10	13.66±1.47

Sample	DHZ	DHZR	DHZOG	DHZROG	DHZ7G	DHZ9G	DHZRMP	Total DHZ-type CKs
**Col-0**	<LOD	0.09±0.02	0.26±0.05	0.11±0.02	3.26±0.40	0.10±0.01	<LOD	3.82±0.45
***gpat4/8***	<LOD	0.08±0.00	0.40±0.06*	0.16±0.02*	3.88±0.65	0.12±0.02	<LOD	4.62±0.73
***gfc1–1***	<LOD	0.09±0.02	0.29±0.03	0.25±0.04**	3.58±0.04	0.13±0.02	<LOD	4.33±0.09

Sample	iP	iPR	iP7G	iP9G	iPRMP	Total iP-type CKs
**Col-0**	4.97±0.62	1.87±0.20	21.97±2.58	2.29±0.03	6.72±1.22	37.83±4.05
***gpat4/8***	3.54±0.83	2.44±0.22*	25.11±1.75	2.73±0.23*	10.72±1.98*	44.53±2.48
***gfc1–1***	1.80±0.30***	1.13±0.25**	20.76±1.08	2.10±0.22	9.70±2.38	35.49±3.91

Sample	Total CK Bases	Total CK Ribosides	Total CK O-glucosides	Total CK N-glucosides	Total CK Nucleotides	Total CKs
**Col-0**	5.42±0.64	3.73±0.42	12.15±1.49	48.10±4.05	15.03±2.64	84.43±8.17
***gpat4/8***	4.13±0.85	4.11±0.22	17.20±0.98**	50.70±3.63	20.29±3.04	96.45±5.66
***gfc1–1***	2.34±0.29***	2.42±0.37**	13.44±2.10	47.19±2.56	20.64±3.24	86.04±7.02

Asterisks indicate statistically significant differences between the mutant lines (*gpat4/8* or *gfc1–1*) and the wild type (Col-0) in an ANOVA analysis. Data are mean of four replicates (*, **, and *** correspond to *P*-values of 0.05 > *P* > 0.01, 0.01 > *P* > 0.001, and *P* < 0.001, respectively). (tZ, cZ, DHZ, iP) R: (tZ, cZ, DHZ, iP) riboside; (tZ, cZ, DHZ) OG: (tZ, cZ, DHZ) O-glucoside; (tZ, cZ, DHZ) ROG: (tZ, cZ, DHZ) riboside O-glucoside; (tZ, DHZ, iP) 7G: (tZ, DHZ, iP) 7-glucoside; (tZ, cZ, DHZ, iP) 9G: (tZ, cZ, DHZ, iP) 9-glucoside; (tZ, cZ, DHZ, iP) RMP: (tZ, cZ, DHZ, iP) riboside-5’-monophosphate; LOD: limit of detection.

## Discussion

### Cutin biosynthetic *GFC1* and *GPAT*s are essential for normal seedling development and CK responses

Our present results reveal that *GFC1/DCR* and at least two *GPATs*, genes encoding acyltransferases that catalyze incorporation of OH-FAs into cutin monomers or polymer [63], significantly influence CK responses and plant development, including skotomorphogenesis in darkness. Previously, *DCR* was identified as a candidate gene whose expression is closely associated with cutin metabolism. Mutation of DCR resulted in many typical phenotypes associated with defective cuticle (Fig. 9C), such as altered epidermal cell differentiation and post genital organ fusion, as well as sensitivity to saline, osmotic, and water stress conditions [37], which we also observed in the T-DNA *gfc1* mutants.

In our present study, we found that in darkness *gfc1* had the de-etiolated phenotypes of short hypocotyls, early opening apical hooks, and overgrown cotyledons (Fig. 5A). Despite its defect in skotomorphogenesis, *gfc1* responded normally to FRc, Rc and Bc light (S5 Fig.), indicating that *GFC1/DCR* is important for skotomorphogenesis in darkness but dispensable for photomorphogenesis under light. Under normal growth conditions, *gfc1* mutant seedlings exhibited stronger staining of the cell cycle reporter *pCYCB1*:*GUS* in root and shoot apexes (Fig. 2F), longer primary root length (Fig. 2A and 2B) and higher fresh weight of shoots (Fig. 2C), suggesting functional roles of *GFC1/DCR* in controlling cell division and differentiation. Pleiotropic mutants with alterations to not only epidermal cuticle integrity but also non-epidermal cell division and differentiation have also been observed in other cuticle mutants [64–66].

*gfc1* was isolated as a CK response mutant in our present study by a forward genetic screen. While the primary roots of *gfc1* responded normally to exogenous CK treatment (Fig. 3A and 3B), the shoots were completely insensitive, even becoming larger in size when treated with CKs (Fig. 3A and 3C). The *gfc1* mutant also showed full insensitivity to CK in darkness, as indicated by hypocotyl length (Fig. 5A-C). Notably, such strong differential responses between *gfc1* and WT were not observed after treatment with IAA, ACC or GA_3_ (Fig. 5D-F), indicating that these *gfc1* phenotypes are CK-specific. Similar to the CK signaling mutant *ahk3*, *gfc1* mutant was less sensitive to CK in adventitious root formation than WT (Fig. 4B). It should be noted that the effect of *gfc1* mutation on its responses to CK in adventitious root production (Fig. 4) was weaker than those on shoots (Fig. 3) or dark-grown hypocotyls (Fig. 5), implying that either *GFC1/DCR* plays limited roles in those processes or its functional loss can be compensated by other related processes.

Although the photomorphogenic phenotype of *gfc1* in darkness (Fig. 5A) implies that the plant should have high endogenous CK levels [55,57,61,62], the mutant did lack other typical high CK phenotypes, such as a bushy appearance. Paradoxically, *gfc1* had longer primary roots with an enlarged MZ, which has been associated with reduced endogenous CK level or signaling [67]. These conflicting phenotypes between *gfc1* and mutants affecting CK levels indicate that the *gfc1* phenotypes are not likely linked to changes in general CK homeostasis. This conclusion is consistent with our CK quantification results (Table 1), in which no causal link was found between levels of active CKs and the *gfc1*-like phenotypes in *gfc1* and *gpat4/8* mutants. Taken together, these results suggest that the *gfc1* mutation leads to defects in CK responses.

In the CK signaling pathway, the type-B *ARR*s positively regulate CK responses by activating the transcription of their downstream targets, including the type-A *ARR* genes. Type-A *ARRs* are rapidly activated in response to exogenous CK and then down-regulate the CK responses through a negative-feedback loop [10]. Consistent with that, multiple loss-of-function mutants in type-A *ARR* genes are hypersensitive to CK in various assays, including inhibition of root elongation, lateral root initiation and callus formation, while over-expression of type-A *ARR* genes can lead to decreased CK sensitivity [20,40]. Our present results show that the basal expression levels of most type-A *ARR* genes were higher in *gfc1*, but that their relative induction levels by exogenous CK were lower than those in WT (Figs. 6, 7A and 7C), Like *gfc1*, *ARR-OX* lines show decreased CK-sensitivity (Fig. 8), which indicates that up-regulation of type-A *ARR*s in *gfc1* and their reduced responses to CK is only part of the reason for the insensitivity of *gfc1* to CK.

### Interaction between CK and ethylene in hypocotyl elongation is disrupted in *gfc1*

**Fig 11:**
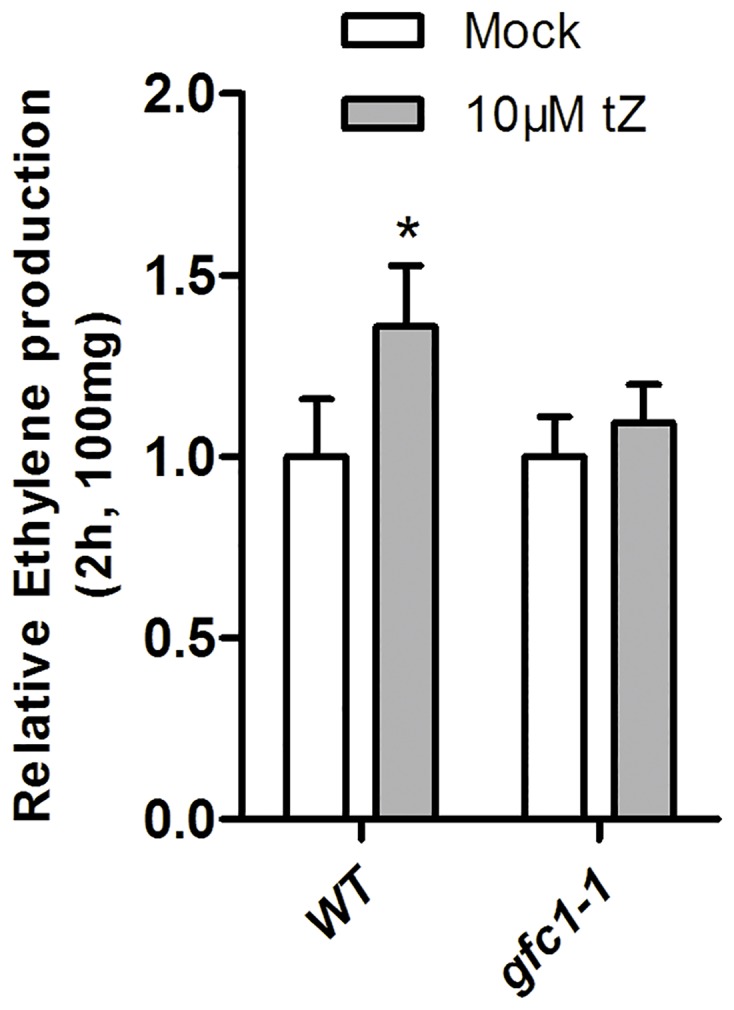
Relative ethylene production. Seedlings were grown in darkness at 5 DAG. Mock-treated seedlings (WT or *gfc1–1* grown on MS) were used as controls. Relative production values = 10μM tZ (WT or *gfc1–1* grown on MS with 10μM tZ) / Mock. Data are mean of four biological replicates. Error bars indicate SD *P <0.05 and **P <0.01 (Student’s t-test) indicate significance between ‘Mock-treated’ and ‘10 μM tZ-treated’.

There is strong cross talk between CK and ethylene in plant growth and development. The application of CK to dark-grown plants can result in the ‘triple response’ that is characteristic of ethylene, and a major part of the effect of CK on root and hypocotyl growth has been reported to be mediated by ethylene [68]. In this process, CK post-transcriptionally increases the activity of the ethylene biosynthesis gene *ACS5*, leading to an elevated level of ethylene biosynthesis [30,31]. We showed that *gfc1* hypocotyls are fully insensitive to CK but positively respond to the ethylene precursor ACC (Fig. 5C and 5D). Consideration of the CK-ethylene cross-talk suggests that signaling from CK to ethylene biosynthesis is disrupted in the *gfc1* mutant. Consistent with that, tZ failed to significantly increase the production of ethylene in dark-grown *gfc1–1* seedlings (Fig. 11).

### Concurrence of *gfc1* phenotypes and blocked acyl-transfer in cutin biosynthesis

Biosynthesis of the epidermal cutin occurs through a complex process that consists of FA synthesis, activation into acyl-CoA, ω- and/or in-chain oxygenation, sn-2 MAG synthesis, monomer/oligomer transport out of the cell to the surface and polymerization into cutin polyester [34]. While the effects of *gfc1/dcr* and *gpat4/8* mutations on cuticle structure can easily be explained by the loss of their respective acyltransferase activities, the biochemical/molecular mechanisms of their effects on CK responses and skotomorphogenesis, which were not reported when the other cuticle mutants were isolated, remain elusive.

The *gfc1/dcr*, *gpat4/8*, *and cyp86A2*/*att1* mutants presented similarly defective cuticles, in terms of decreased cutin monomer loads and increased permeability to solutes (Fig. 9C), but *cyp86A2*/*att1* had normal CK responses and skotomorphogenesis (Fig. 9A and 9B), indicating that the phenotypes of *gfc1* and *gpat4/8* are not simply due to the lack of an intact cuticle layer in the epidermis. Cuticle mutants often display pleiotropic phenotypes, some of which appear to be not directly linked to their primary effects on cuticle integrity, such as alteration to non-epidermal cell development [37,64–66]. Another example is the sensitivity to pathogen infection. Xiao et al. (2004) reported that *Arabidopsis CYP86A2/ATT1* is required for cuticle development and can represses *Pseudomonas syringae* type III genes and that *cyp86A2/att1*, but not *wax2* mutation, could lead to enhanced avrPto-luc expression. Both mutants had similar cuticle defects, but *cyp86A2/att1* had an additional gene-specific but not cuticle-specific effect. They suggested that certain cutin-related FAs synthesized by CYP86A2 may repress bacterial type III gene expression [69].

GPAT4/8 catalyzes the formation of sn2-OH-MAG, one of the major monomers of cutin, by sn-2-specific G3P: acyl-CoA acyltransferase as well as phosphatase activities [70]. Although the natural substrate and product of GFC1/DCR *in vivo* is still unknown [34], it has an *in vitro* diacylglycerol acyltransferase activity [38]. GFC1/DCR may function in acyltransfer of cutin monomers to form precursor intermediates or oligomeric structures [37]. With free—OH group, tZ is likely to be a substrate of these acyltransferases. However, such hypothesis conflicts with the similar effects of tZ and other CKs without free—OH group on *gfc1* and *gpat4/8* (S3 Fig.). As a P450 monooxgenase, CYP86A2/ATT1 has been shown to be associated with the ω-hydroxylation of FA [69] and the production of α,ω-dicarboxylic acid (DCA) [71]. Therefore, *gfc1* phenotypes are produced by blocking the GPATs or/and GFC1/DCR catalyzed acyl-transfer but not by preventing the *CYP86A2*-mediated FA ω-hydroxylation steps in the cutin biosynthesis pathway.

Hydroxylation of FAs by CYP86As, transfer of the OH-FA to acylate G3P by GPATs, and probably their further acylation into cutin polymer by GFC1/DCR are closely linked in the core reaction of cutin biosynthesis [34], making it likely that *cyp86A2/att1* has decreased OH-FAs levels and *gfc1/dcr* and *gpat4/8* have increased OH-FAs. The involvement of OH-FAs as constituents of membrane lipids, and as biosynthetic precursors for biologically active compounds such as jasmonates, and the existence of OH-FA-dependent signaling in plant cells, have been noted previously in the literature [64].

Cutin biosynthesis is complex and its organization and regulation remain largely uncertain. It is unknown how *gfc1* and *gpat4/8* link lipid metabolism or signaling pathways, cutin-associated acyltransferase, and CK response and skotomorphogenesis. One possibility is that the accumulation of OH-FAs may perturb cell membranes and affect CK responses. More studies at the biochemical, molecular, physiological and genetic levels are needed to uncover the mechanisms underlying the roles of acyltransferases in CK responses and skotomorphogenesis.

## Supporting Information

S1 Appendix T-DNA flanking sequence in *gfc1–1*. (DOC)

S2 Appendix PCR primer sequences. (DOC)

S3 Appendix Genotyping primers for mutant lines. (DOC)

S1 Fig Gene structures, mutations, and RT-PCR analysis of *GFC1/DCR*, *GPAT4*, *GPAT8* and *CYP86A2*. (A) Gene structures and mutations. The primers in figure were used for RT-PCR. (B) RT-PCR analysis (30 cycles) of gene transcripts. The *ACT-2* was used as a control.
(TIF)

S2 Fig Percentage of non-germinating seeds of WT and *gfc1* on MS plates 5 DAG. Data are means of three biological replicates, n>80. Error bars indicate SD. *P <0.05 and **P <0.01 (Student’s t-test) indicate significant differences between ‘WT’ and ‘*gfc1–1* or *gfc1–2*’.
(TIF)

S3 Fig Phenotypes of WT and *gfc1–1* seedlings grown on MS plates supplemented with different CKs. (A-C) Long day conditions, 7 DAG. (D-E) Darkness, 5 DAG. (B, C, E) All the data are means of three biological replicates, n = 25. Error bars indicate SD. *P <0.05 and **P <0.01 (Student’s t-test) indicating significant difference between ‘WT’ and ‘*gfc1–1*’.
(TIF)

S4 Fig Phenotypes of WT and *gfc1–1* seedlings grown on MS plates supplemented with different hormones. (A-C) Long day conditions, 7 DAG. (B, C) All the data are mean of three biological replicates, n = 25. Error bars indicate SD. *P <0.05 and **P <0.01 (Student’s t-test) indicating significant differences between ‘WT’ and ‘*gfc1–1*’.
(TIF)

S5 Fig Responses of WT, *gfc1–1*, *phyA*, and *phyB* seeds to FRc, Rc and Bc light. (A) 5 DAG. (B-C) Absolute (B) and relative (C) hypocotyl length. WT was used as a control. Data are means of three biological replicates, n>20. Error bars indicate SD. *P <0.05 and **P <0.01 (Student’s t-test) indicating significant differences between ‘WT’ and ‘mutants’.
(TIF)

S6 Fig Comparison of CK-induced phenotypes in WT, *gpat4*, *gpat8*, *gfc1–1*, *gpat4/8* and *gfc1–1/gpat4/8*. (A, C) Darkness, 5 DAG. (B, D) Long day conditions, 7 DAG. (C, D) WT was used as a control. Data are means of three biological replicates, n>20. Error bars indicate SD. *P <0.05 and **P <0.01 (Student’s t-test) indicating significant differences between ‘WT’ and ‘mutants’.
(TIF)

S7 Fig Comparison of CK-induced phenotypes in WT, *gfc1–1* and *cyp86A2*. (A, C) Darkness, 5 DAG. (B, D) Long day conditions, 7 DAG. (C, D) WT was used as a control. Data are means of three biological replicates, n>20. Error bars indicate SD. *P <0.05 and **P <0.01 (Student’s t-test) indicating significant differences between ‘WT’ and ‘mutants’.
(TIF)
