# Supplementary material for: Functional Roles of Three Cutin Biosynthetic Acyltransferases in Cytokinin Responses and Skotomorphogenesis
Source: PLoS One. 2015 Mar 24;10(3):e0121943. doi: 10.1371/journal.pone.0121943 (PMC4372371; doi:10.1371/journal.pone.0121943)
Supplement: S1 Appendix — (DOC) [file pone.0121943.s001.doc]

**S1 Appendix.** T-DNA flanking sequences in *gfc1-1.*

**TTGCCCGTCTCACTGGTGAAAAGAAAAACCACCCCAGTACATTAAAAACGTCCGCAATGTGTTATTAAGTTGTCTAAGCGTCAATTTGTTTACACCACAAATTACACAATAAAAAAAAAAAACAGTAAAAAAAAAGGGATTCAAAAACCGAGTTTCCCAAATCATTGACTACAATTACCGTAAAGATGAAAATGAACATACACATATTTCTAAAACATATGTGTATGGTCATGGTATAATACGTGCATGTGTAATATATTCATAAGCATAAAGTATGCATGT**

**pROK2 T-DNA LB sequence; gene AT5g23940 sequence**

**ACTCCCTTAATTCTCCGCTCATGATCAGATTGTCGTTTCCCGCCTTCAGTTTAAACTATCAGTGTTTGACAGGATATATTGGCGGGTAAACCTAAGAGAAAAGAGCGTTTATTAGAATAAactgaccaataacatgctacggatacaaactatgagtaaatagcaaacaccacgtatgcatgtacaaaataagtagatagattaaatttgtatatatcactcaacaaatagtacacattagagaatttaatagtcattatcaatcaatcctatacaaatataaatatcagctttagcgc**

**pROK2 T-DNA RB sequence; gene AT5g23940 sequence**
