# Supplementary material for: Functional Roles of Three Cutin Biosynthetic Acyltransferases in Cytokinin Responses and Skotomorphogenesis
Source: PLoS One. 2015 Mar 24;10(3):e0121943. doi: 10.1371/journal.pone.0121943 (PMC4372371; doi:10.1371/journal.pone.0121943)
Supplement: S2 Appendix — (DOC) [file pone.0121943.s002.doc]

**S2 Appendix.** PCR primer sequences.

P1: TTCCTTACCTTGCTTTCTACT

P2: TTCTTTCCATCATCTTCTTCT

P3: TGAACTTAGCCGTCCCGTCCT

QRT-ACT8-F: TGTGACAATGGTACTGGAATGG

QRT-ACT8-R: TTGGATTGTGCTTCATCACC

QRT-GFC-F: GATCGACGCACGTAACGAC

QRT-GFC-R: CAAACCCGAAATCCACCTC

QRT-ARR3-F: GCCGTGACTTTGCAGGATGTGATT

QRT-ARR3-R: TCTCGCCGTTGATGACAGCCTA

QRT-ARR4-F: TCCTGCAAAGTTACGGCGGTA

QRT-ARR4-R: AACCAGTCATTCCAGGCATACAGT

QRT-ARR5-F: CCAGTCATCCCAGGCATAGAGT

QRT-ARR5-R: GATCGGAAGTTCATCGAGCGG

QRT-ARR6-F: GTCGGTTTTGAGGATTTGAAGGTT

QRT-ARR6-R: TCATACCGGGCATTGAGTAATCAG

QRT-ARR7-F: TCCTGAAAGTCCTGGCATTGAGTA

QRT-ARR7-R: AAGTGACGACTGTAGAGAGTGGAA

QRT-ARR8-F: ACTCTCTACATCGCCACAAA

QRT-ARR8-R: TCTAAACGCCGCTGATTCCT

QRT-ARR9-F: TTCCTGCAAGAATCAGCAGATGTT

QRT-ARR9-R: AGTTGTCTCAATCTCCTCCAGCTT

QRT-ARR15-F: GGAGTGTCGTCATCAAGGGAG

QRT-ARR15-R: GAGGTGGTGAAGCTGAAGAAGG

QRT-ARR16-F: GCGCATTCTCTGCTGTTGTCACTT

QRT-ARR16-R: CCGTAAACTCGTTGAGAGGTTGCT

QRT-ARR17-F: GCCAGGAATGACAGGTTTTGAGC

QRT-ARR17-R: TCGCTAAACATTTGTTGATGCGGG

RT-ARR3-F: TGTCGTCGGAGAATGTAATGA

RT-ARR3-R: AGATTCCATCGAGGATGTGG

RT-ARR6-F: CGGTATGACTGGATATGAAC

RT-ARR6-R: ATTTGCATCGGAGAGCTC

Actin2-F: GTTGGGATGAACCAGAAGGA

Actin2-R: CTTACAATTTCCCGCTCTGC

PGFC1-F2: GCTGCAGGTTGTCTTGCGATATGCTTGTG (Pst I)

PGFC1-R: CGGGATCCGGTGAAAGAGATTTTAACTGGC (BamH I)

GFC1-F: CGGGATCCCACAATACCACATTGATCCAAGG (BamH I)

GFC1-R: GGGGTACCTCAAACAAACCCATTGCCATTTC (Kpn I)

GFC1-2R: GCGGATCCAACAAACCCATTGCCATTTCC (BamH I)

3362-F: CATCTCCTAACTACTGAAGAGTGC

3362-R: ACGAATCTCAATATTACACGACG

salk_128228c-F: ATCCACGTGGCATTTTATGAG

salk_128228c-R: ACAATTCCAAACCAAACACAC

salk_106893-F: TCTCTTCCCATCGTCATCATC

salk_106893-R: ACTGTTGTGGCTGATTTGGTC

GPAT4 RT-F: CCTTCCCTCTCTTTCTTTCC

salk_035914-F: AAACTCCTATTGAACCCCCTG

salk_035914-R: TAACGAATCACCTGATCTCGG

GPAT8 RT-F: TTCTCATGTTGGCAGCATCG

salk_128714c-F: GGTTCATGGGATGATGTGTTC

salk_128714c-R: GCTCCGTTCTGATTGAGACAC

cyp86A2 RT-F: AATGGTTCGCTGGTCTGTTTCAT

ahk3-1-F: GTATCTCGGGGGAGTGTTTGACATT

ahk3-1-R: TGTGACTTTGCAACATCTGCTGCTT

J202: CATTTTATAATAACGCTGCGGACATCTAC

LBa_1_: TGGTTCACGTAGTGGGCCATCG

LBb_1.3_: ATTTTGCCGATTTCGGAAC

LB_6313_: TCAAACAGGATTTTCGCCTGCT

LBb_1_: GCGTGGACCGCTTGCTGCAACT

P9: TTGAACAAGATGGATTGCACG

P10: GATGACAGGAGATCCTGCCC

Z1: GCGAGAAAGGAAGGGAAGAAAGC

Z2: ATAATCATCGCAAGACCGGCAAC

Z3: ATTTCCTGGAGATTATTGCTCGGG

Z4: CTTTGACTCCATGGCCTTTGATTC
