# Supplementary material for: Functional Roles of Three Cutin Biosynthetic Acyltransferases in Cytokinin Responses and Skotomorphogenesis
Source: PLoS One. 2015 Mar 24;10(3):e0121943. doi: 10.1371/journal.pone.0121943 (PMC4372371; doi:10.1371/journal.pone.0121943)
Supplement: S3 Appendix — (DOC) [file pone.0121943.s003.doc]

**S3 Appendix. Genotyping primers for mutant lines.**

| **Allele** | **Origin** | **Mutant fragment primers** | **WT fragment primers** |
| --- | --- | --- | --- |
| ***gfc1-1*** |  | 3362-F + LBb_1.3_ | 3362-F + 3362-R |
| ***gfc1-2*** | salk_128228c | salk_128228-R + LBb_1.3_ | salk_128228-R + salk_128228-F |
| ***ahk3-1*** | N6562 | ahk3-1-R + J202 | ahk3-1-R + ahk3-1-F |
| ***gpat4*** | salk_106893 | salk_106893-R + LBb_1.3_ | salk_106893-R + salk_106893-F |
| ***gpat8*** | salk_035914 | salk_035914-R + LBb_1.3_ | salk_035914-R + salk_035914-F |
| ***cyp86A2*** | salk_128714c | salk_128714c-R + LBb_1.3_ | salk_128714c-R + salk_128714c-F |
