# Supplementary figures and images for: Functional Roles of Three Cutin Biosynthetic Acyltransferases in Cytokinin Responses and Skotomorphogenesis
Source: PLoS One. 2015 Mar 24;10(3):e0121943. doi: 10.1371/journal.pone.0121943 (PMC4372371; doi:10.1371/journal.pone.0121943)

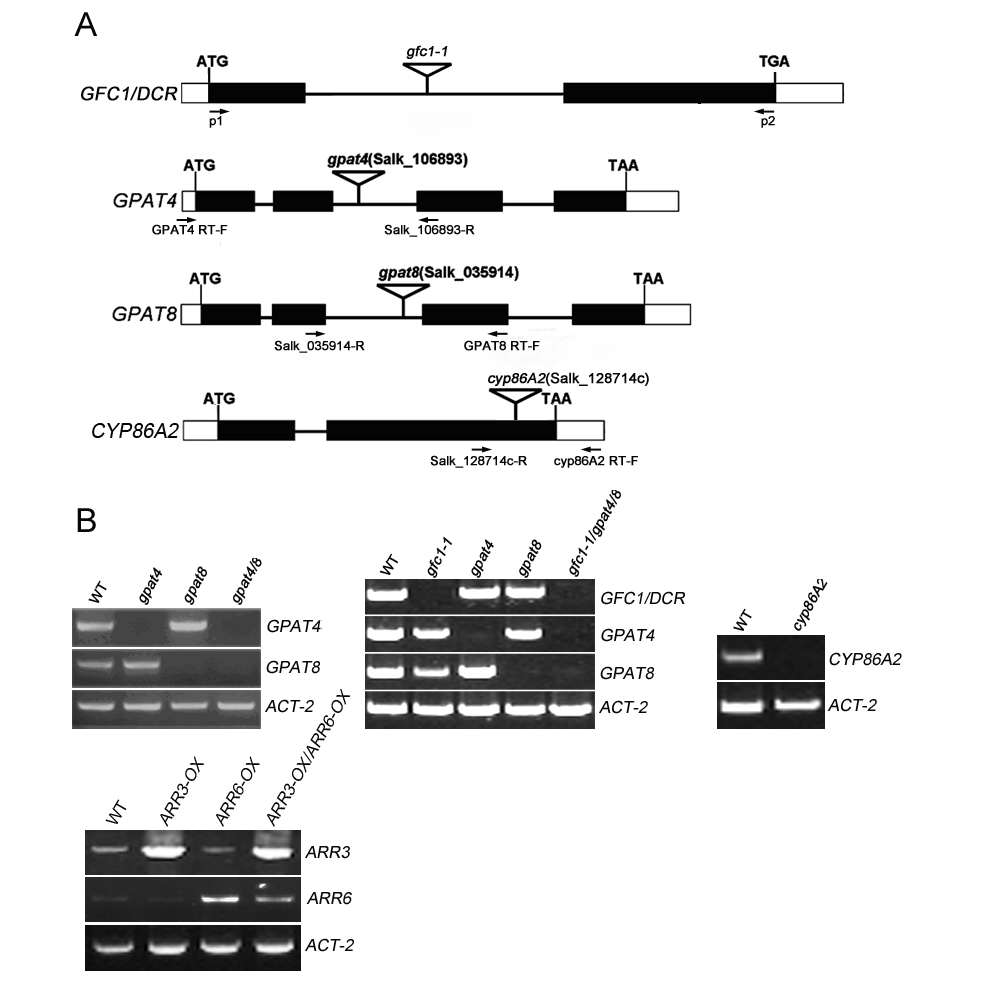

Supplement: S1 Fig — (A) Gene structures and mutations. The primers in figure were used for RT-PCR. (B) RT-PCR analysis (30 cycles) of gene transcripts. The ACT-2 was used as a control. (TIF) [file pone.0121943.s004.tif]

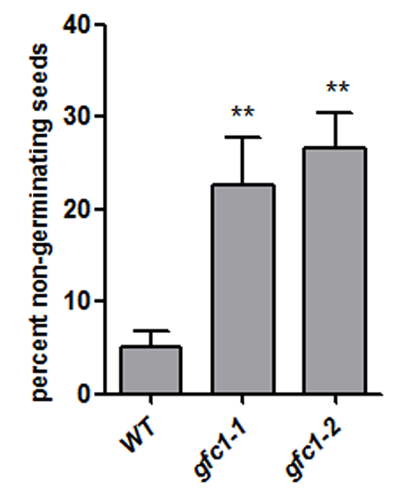

Supplement: S2 Fig — Data are means of three biological replicates, n>80. Error bars indicate SD. *P <0.05 and **P <0.01 (Student’s t-test) indicate significant differences between ‘WT’ and ‘gfc1–1 or gfc1–2’. (TIF) [file pone.0121943.s005.tif]

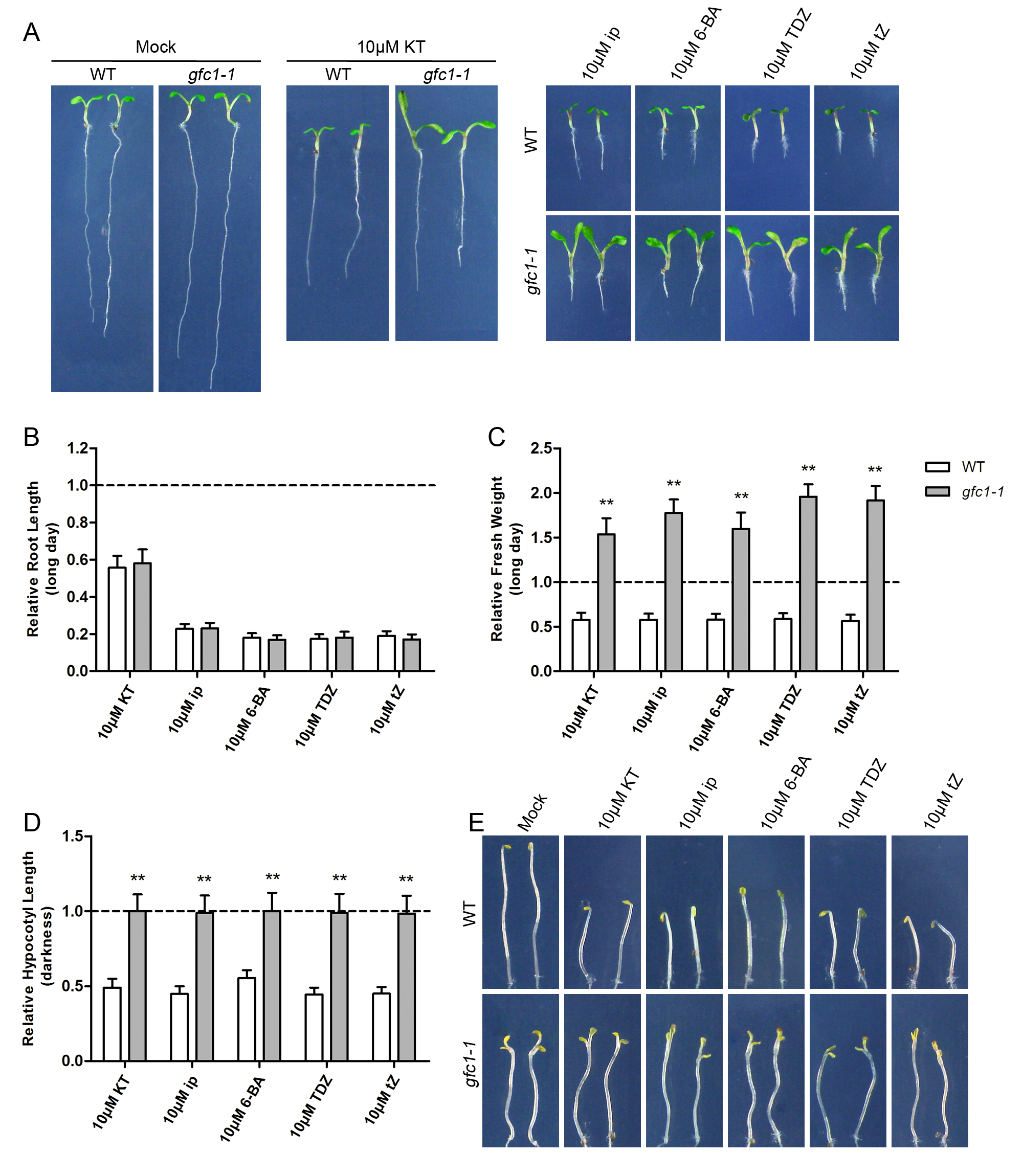

Supplement: S3 Fig — (A-C) Long day conditions, 7 DAG. (D-E) Darkness, 5 DAG. (B, C, E) All the data are means of three biological replicates, n = 25. Error bars indicate SD. *P <0.05 and **P <0.01 (Student’s t-test) indicating significant difference between ‘WT’ and ‘gfc1–1’. (TIF) [file pone.0121943.s006.tif]

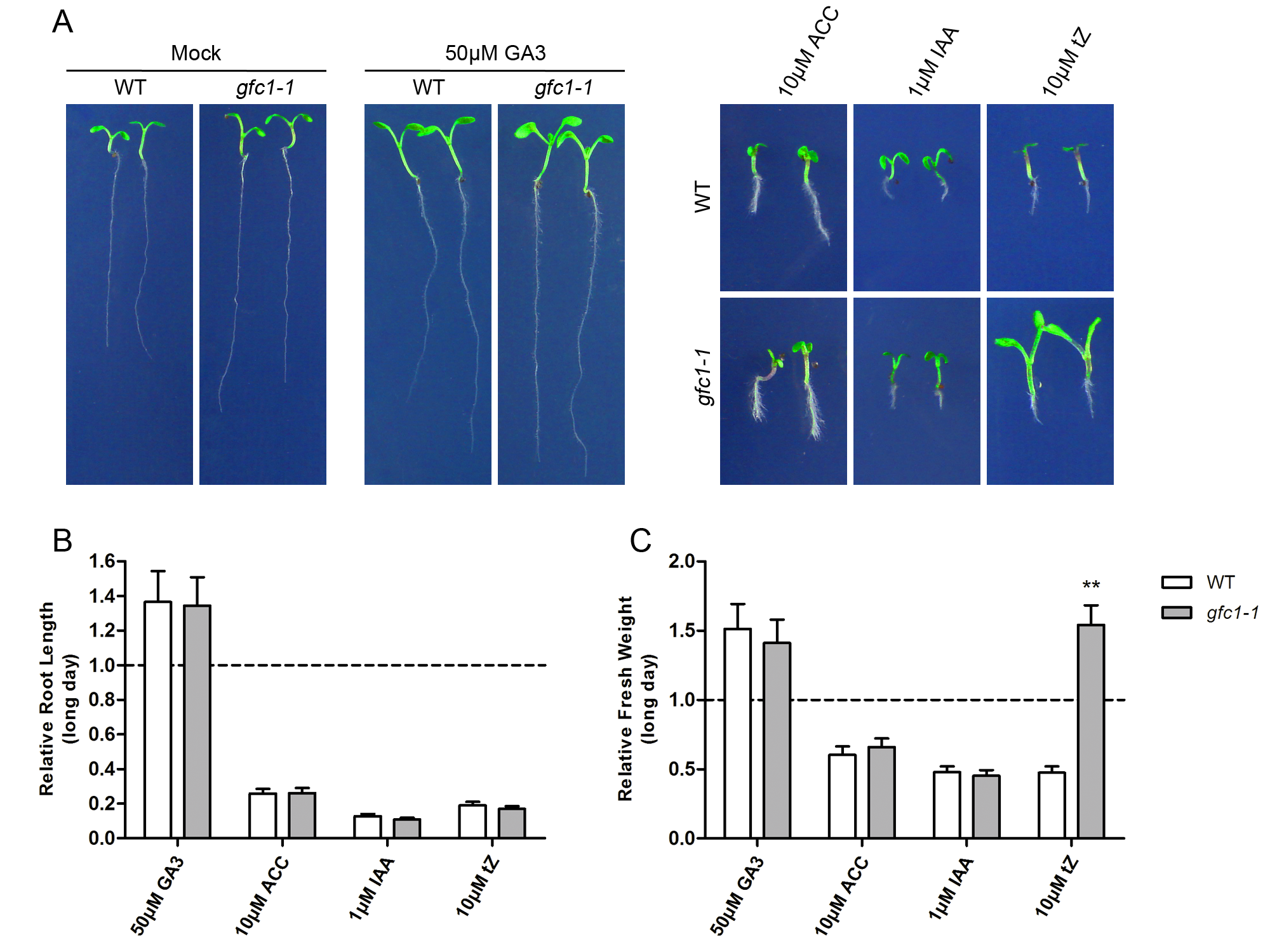

Supplement: S4 Fig — (A-C) Long day conditions, 7 DAG. (B, C) All the data are mean of three biological replicates, n = 25. Error bars indicate SD. *P <0.05 and **P <0.01 (Student’s t-test) indicating significant differences between ‘WT’ and ‘gfc1–1’. (TIF) [file pone.0121943.s007.tif]

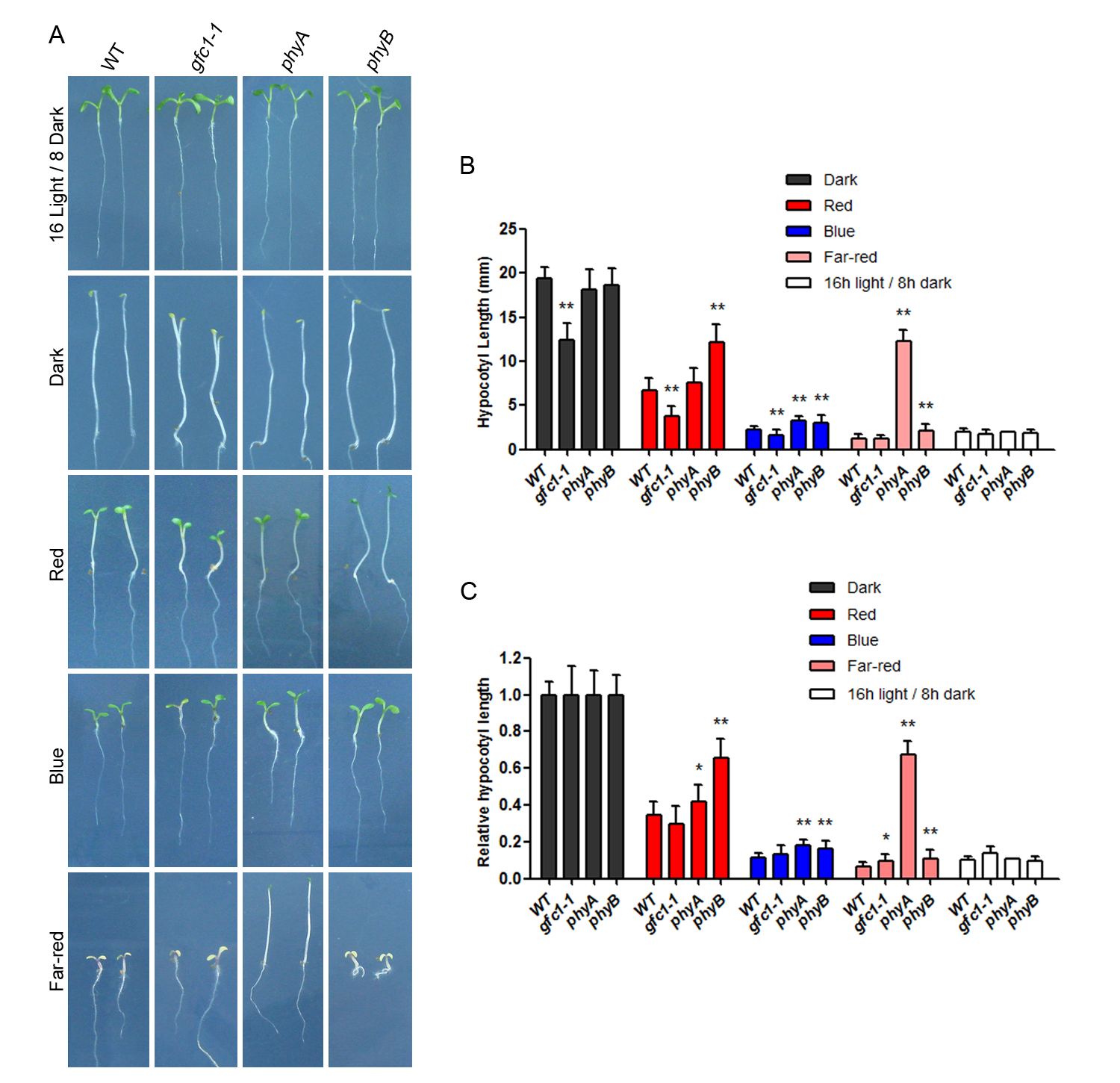

Supplement: S5 Fig — (A) 5 DAG. (B-C) Absolute (B) and relative (C) hypocotyl length. WT was used as a control. Data are means of three biological replicates, n>20. Error bars indicate SD. *P <0.05 and **P <0.01 (Student’s t-test) indicating significant differences between ‘WT’ and ‘mutants’. (TIF) [file pone.0121943.s008.tif]

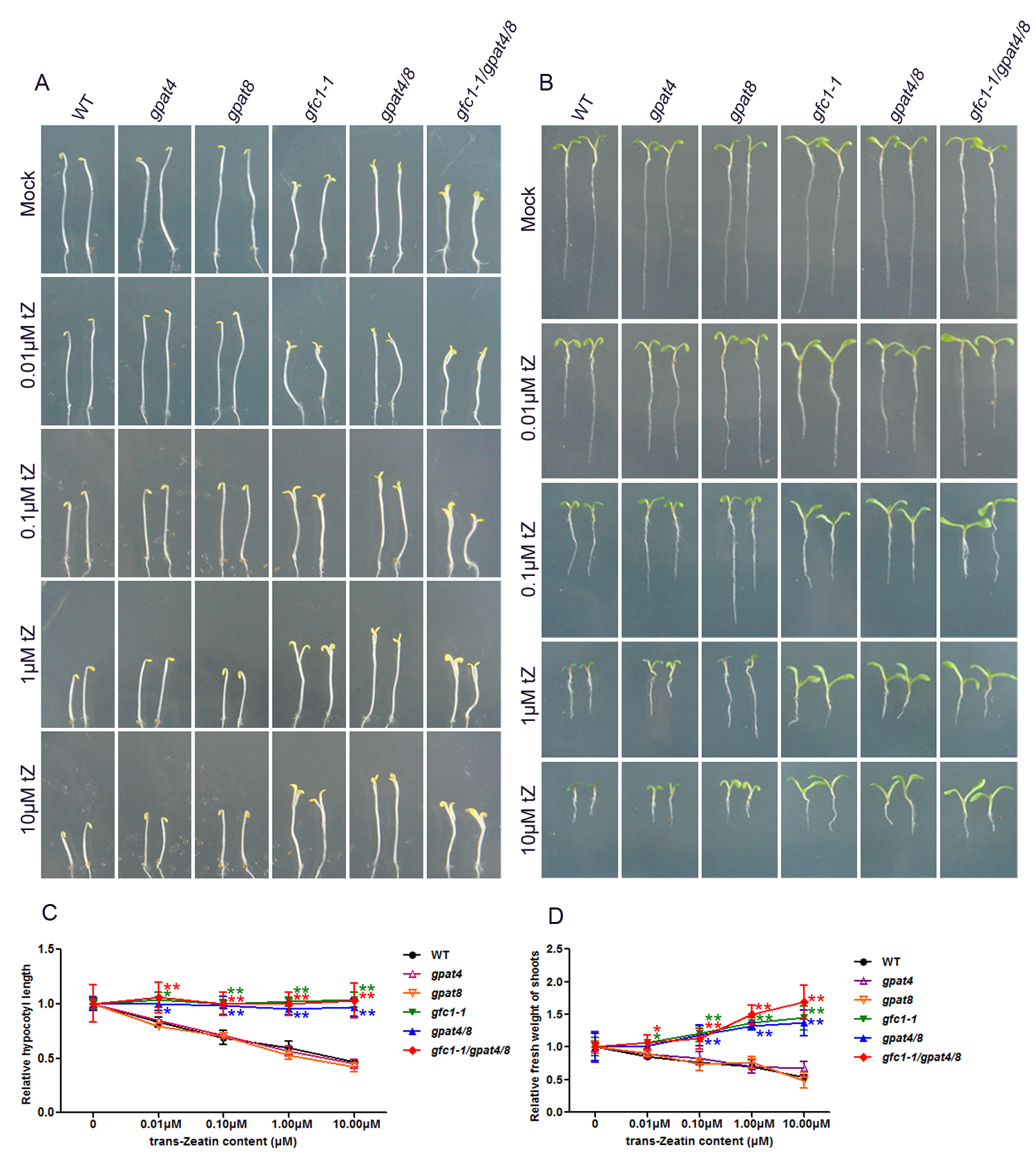

Supplement: S6 Fig — (A, C) Darkness, 5 DAG. (B, D) Long day conditions, 7 DAG. (C, D) WT was used as a control. Data are means of three biological replicates, n>20. Error bars indicate SD. *P <0.05 and **P <0.01 (Student’s t-test) indicating significant differences between ‘WT’ and ‘mutants’. (TIF) [file pone.0121943.s009.tif]

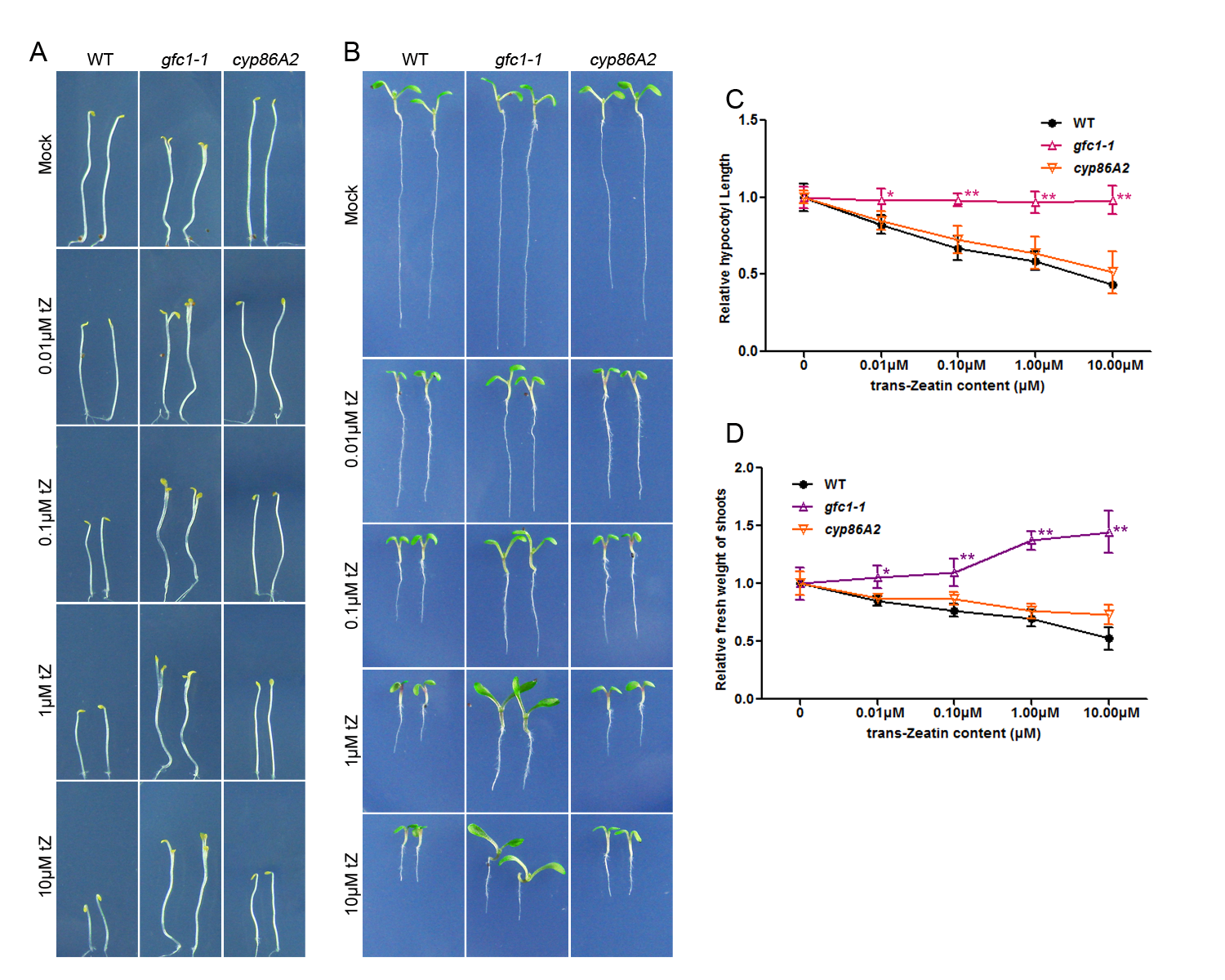

Supplement: S7 Fig — (A, C) Darkness, 5 DAG. (B, D) Long day conditions, 7 DAG. (C, D) WT was used as a control. Data are means of three biological replicates, n>20. Error bars indicate SD. *P <0.05 and **P <0.01 (Student’s t-test) indicating significant differences between ‘WT’ and ‘mutants’. (TIF) [file pone.0121943.s010.tif]
